# 4-Chloro-2-[(*E*)-2-(4-methoxy­phen­yl)ethyl­imino­meth­yl]phenol

**DOI:** 10.1107/S1600536809013348

**Published:** 2009-04-18

**Authors:** Marzieh Yaeghoobi, Noorsaadah Abdul Rahman, Seik Weng Ng

**Affiliations:** a Department of Chemistry, University of Malaya, 50603 Kuala Lumpur, Malaysia

## Abstract

In the title Schiff base, C_16_H_16_ClNO_2_, the 2-(4-methoxy­phen­yl)ethyl (CH_3_OC_6_H_4_CH_2_CH_2_–; r.m.s. deviation = 0.10 Å) and 4-chloro-2-(imino­meth­yl)phenol (N=CHC_6_H_3_ClOH; r.m.s. deviation = 0.01 Å) portions are both essentially planar, the two parts being inclined at an angle of 61.8 (1)°. The hydroxy group forms a hydrogen bond to the imino N atom.

## Related literature

The crystal structures of several Schiff bases derived by condensing aryl-2-ethyl­amines and substituted salicylaldehydes have been reported; see: Chatziefthimiou *et al.* (2006 ▶); Chohan *et al.* (2008 ▶); Coombs *et al.* (2005 ▶); Li *et al.* (2006 ▶); Räisänen *et al.* (2007 ▶).
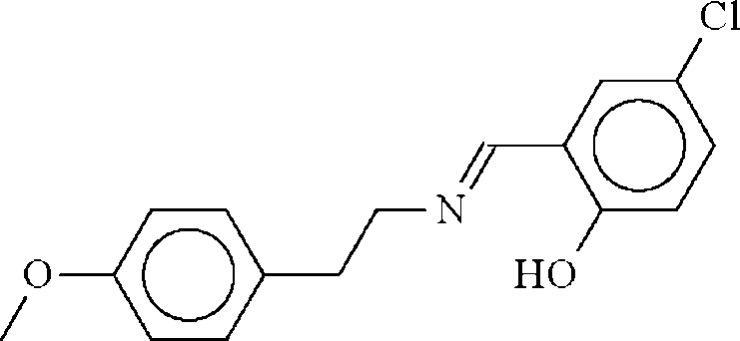


## Experimental

### 

#### Crystal data

C_16_H_16_ClNO_2_
*M* *_r_* = 289.75
Triclinic, 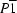

*a* = 5.7610 (2) Å
*b* = 7.7115 (3) Å
*c* = 15.7814 (5) Å
α = 82.420 (2)°
β = 89.320 (2)°
γ = 85.313 (2)°
*V* = 692.65 (4) Å^3^
*Z* = 2
Mo *K*α radiation
μ = 0.28 mm^−1^
*T* = 100 K
0.25 × 0.25 × 0.03 mm

#### Data collection

Bruker SMART APEX diffractometer
Absorption correction: multi-scan (*SADABS*; Sheldrick, 1996 ▶) *T*_min_ = 0.934, *T*_max_ = 0.992
5284 measured reflections
3036 independent reflections
2235 reflections with *I* > 2σ(*I*)
*R*_int_ = 0.025

#### Refinement

*R*[*F* ^2^ > 2σ(*F* ^2^)] = 0.040
*wR*(*F* ^2^) = 0.099
*S* = 1.03
3036 reflections
186 parameters
1 restraint
H atoms treated by a mixture of independent and constrained refinement
Δρ_max_ = 0.28 e Å^−3^
Δρ_min_ = −0.28 e Å^−3^

### 

Data collection: *APEX2* (Bruker, 2008 ▶); cell refinement: *SAINT* (Bruker, 2008 ▶); data reduction: *SAINT*; program(s) used to solve structure: *SHELXS97* (Sheldrick, 2008 ▶); program(s) used to refine structure: *SHELXL97* (Sheldrick, 2008 ▶); molecular graphics: *X-SEED* (Barbour, 2001 ▶); software used to prepare material for publication: *publCIF* (Westrip, 2009 ▶).

## Supplementary Material

Crystal structure: contains datablocks global, I. DOI: 10.1107/S1600536809013348/sj2612sup1.cif

Structure factors: contains datablocks I. DOI: 10.1107/S1600536809013348/sj2612Isup2.hkl

Additional supplementary materials:  crystallographic information; 3D view; checkCIF report

## Figures and Tables

**Table 1:** Hydrogen-bond geometry (Å, °)

*D*—H⋯*A*	*D*—H	H⋯*A*	*D*⋯*A*	*D*—H⋯*A*
O1—H1⋯N1	0.85 (1)	1.79 (2)	2.567 (2)	152 (3)

**Fig. 1.**
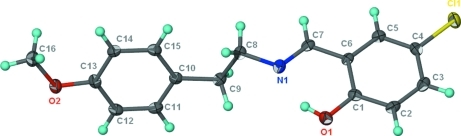
Thermal ellipsoid plot of C16H16ClNO2 at the 70% probability level; hydrogen atoms are drawn as spheres of arbitrary radius.

## supplementary crystallographic information

### Experimental

2-(4-Methoxyphenyl)ethylamine (0.30 g, 2 mmol) and 5-chlorosalicylaldehyde (0.31 g, 2 mmol) were heated in ethanol (20 ml) for 1 h. The solution was set aside for the growth of crystals.

### Refinement

Carbon-bound H-atoms were placed in calculated positions (C—H 0.95 to 0.99 Å) and were included in the refinement in the riding model approximation, with *U*(H) fixed at 1.2–1.5*U*(C).

The hydroxy H-atom was located in a difference Fourier map, and was refined with a distance restraint of O–H 0.84±0.01 Å; its temperature factor was refined.

### Crystal data

**Table:**

C_16_H_16_ClNO_2_	*Z* = 2
*M**_r_* = 289.75	*F*(000) = 304
Triclinic, *P*1	*D*_x_ = 1.389 Mg m^−^^3^
Hall symbol: -P 1	Mo *K*α radiation, λ = 0.71073 Å
*a* = 5.7610 (2) Å	Cell parameters from 1628 reflections
*b* = 7.7115 (3) Å	θ = 2.7–28.2°
*c* = 15.7814 (5) Å	µ = 0.28 mm^−^^1^
α = 82.420 (2)°	*T* = 100 K
β = 89.320 (2)°	Plate, yellow
γ = 85.313 (2)°	0.25 × 0.25 × 0.03 mm
*V* = 692.65 (4) Å^3^	

### Data collection

**Table:**

Bruker SMART APEX diffractometer	3036 independent reflections
Radiation source: fine-focus sealed tube	2235 reflections with *I* > 2σ(*I*)
graphite	*R*_int_ = 0.025
ω scans	θ_max_ = 27.5°, θ_min_ = 2.6°
Absorption correction: multi-scan (*SADABS*; Sheldrick, 1996)	*h* = −7→7
*T*_min_ = 0.934, *T*_max_ = 0.992	*k* = −10→9
5284 measured reflections	*l* = −20→20

### Refinement

**Table:**

Refinement on *F*^2^	Primary atom site location: structure-invariant direct methods
Least-squares matrix: full	Secondary atom site location: difference Fourier map
*R*[*F*^2^ > 2σ(*F*^2^)] = 0.040	Hydrogen site location: inferred from neighbouring sites
*wR*(*F*^2^) = 0.099	H atoms treated by a mixture of independent and constrained refinement
*S* = 1.03	*w* = 1/[σ^2^(*F*_o_^2^) + (0.033*P*)^2^ + 0.4582*P*] where *P* = (*F*_o_^2^ + 2*F*_c_^2^)/3
3036 reflections	(Δ/σ)_max_ = 0.001
186 parameters	Δρ_max_ = 0.28 e Å^−^^3^
1 restraint	Δρ_min_ = −0.28 e Å^−^^3^

### Fractional atomic coordinates and isotropic or equivalent isotropic displacement parameters (Å^2^)

**Table:**

	*x*	*y*	*z*	*U*_iso_*/*U*_eq_	
Cl1	0.72711 (10)	0.19401 (7)	0.02737 (3)	0.02303 (15)	
O1	0.4122 (3)	0.0304 (2)	0.38188 (9)	0.0209 (3)	
H1	0.509 (4)	0.068 (4)	0.4137 (15)	0.042 (8)*	
O2	0.9991 (2)	0.43513 (19)	0.88357 (8)	0.0184 (3)	
N1	0.7683 (3)	0.1729 (2)	0.42911 (10)	0.0157 (4)	
C1	0.4895 (3)	0.0673 (3)	0.30093 (12)	0.0147 (4)	
C2	0.3616 (3)	0.0202 (3)	0.23431 (13)	0.0168 (4)	
H2	0.2243	−0.0389	0.2465	0.020*	
C3	0.4334 (4)	0.0592 (3)	0.15057 (13)	0.0177 (4)	
H3	0.3449	0.0282	0.1053	0.021*	
C4	0.6357 (4)	0.1440 (3)	0.13294 (12)	0.0161 (4)	
C5	0.7667 (3)	0.1895 (3)	0.19782 (12)	0.0150 (4)	
H5	0.9056	0.2460	0.1847	0.018*	
C6	0.6960 (3)	0.1530 (2)	0.28272 (12)	0.0130 (4)	
C7	0.8351 (3)	0.2023 (3)	0.35138 (12)	0.0139 (4)	
H7	0.9757	0.2564	0.3381	0.017*	
C8	0.9076 (3)	0.2214 (3)	0.49754 (12)	0.0157 (4)	
H8A	1.0524	0.2691	0.4737	0.019*	
H8B	0.9509	0.1165	0.5393	0.019*	
C9	0.7660 (3)	0.3591 (3)	0.54177 (12)	0.0159 (4)	
H9A	0.7698	0.4739	0.5056	0.019*	
H9B	0.6019	0.3293	0.5451	0.019*	
C10	0.8459 (3)	0.3790 (3)	0.63111 (12)	0.0142 (4)	
C11	0.6936 (3)	0.4703 (3)	0.68291 (13)	0.0158 (4)	
H11	0.5482	0.5219	0.6603	0.019*	
C12	0.7501 (4)	0.4870 (3)	0.76572 (13)	0.0163 (4)	
H12	0.6443	0.5498	0.7995	0.020*	
C13	0.9625 (4)	0.4120 (3)	0.80025 (12)	0.0159 (4)	
C14	1.1189 (4)	0.3236 (3)	0.75010 (12)	0.0152 (4)	
H14	1.2652	0.2739	0.7726	0.018*	
C15	1.0580 (3)	0.3086 (3)	0.66572 (13)	0.0156 (4)	
H15	1.1654	0.2486	0.6314	0.019*	
C16	1.2113 (4)	0.3563 (3)	0.92316 (13)	0.0212 (5)	
H16A	1.2147	0.3805	0.9825	0.032*	
H16B	1.3442	0.4054	0.8920	0.032*	
H16C	1.2202	0.2292	0.9220	0.032*	

### Atomic displacement parameters (Å^2^)

**Table:**

	*U*^11^	*U*^22^	*U*^33^	*U*^12^	*U*^13^	*U*^23^
Cl1	0.0271 (3)	0.0286 (3)	0.0145 (2)	−0.0072 (2)	0.0018 (2)	−0.0036 (2)
O1	0.0214 (8)	0.0262 (9)	0.0161 (7)	−0.0088 (7)	0.0029 (6)	−0.0026 (6)
O2	0.0195 (8)	0.0215 (8)	0.0141 (7)	−0.0005 (6)	−0.0014 (6)	−0.0023 (6)
N1	0.0174 (9)	0.0134 (9)	0.0164 (8)	−0.0008 (7)	−0.0022 (7)	−0.0024 (7)
C1	0.0144 (10)	0.0119 (10)	0.0172 (10)	0.0019 (8)	0.0020 (8)	−0.0011 (8)
C2	0.0130 (10)	0.0151 (10)	0.0226 (11)	−0.0015 (8)	−0.0012 (8)	−0.0033 (8)
C3	0.0171 (10)	0.0153 (10)	0.0211 (10)	−0.0005 (8)	−0.0045 (8)	−0.0046 (8)
C4	0.0181 (11)	0.0159 (10)	0.0139 (9)	0.0008 (8)	0.0014 (8)	−0.0016 (8)
C5	0.0144 (10)	0.0118 (10)	0.0185 (10)	−0.0008 (8)	−0.0001 (8)	−0.0011 (8)
C6	0.0121 (10)	0.0108 (10)	0.0157 (9)	0.0014 (8)	−0.0030 (8)	−0.0014 (8)
C7	0.0116 (10)	0.0109 (10)	0.0190 (10)	−0.0001 (8)	−0.0017 (8)	−0.0011 (8)
C8	0.0167 (10)	0.0141 (10)	0.0162 (10)	−0.0007 (8)	−0.0019 (8)	−0.0020 (8)
C9	0.0158 (10)	0.0136 (10)	0.0182 (10)	−0.0010 (8)	−0.0019 (8)	−0.0022 (8)
C10	0.0149 (10)	0.0112 (10)	0.0167 (10)	−0.0041 (8)	0.0014 (8)	−0.0010 (8)
C11	0.0119 (10)	0.0133 (10)	0.0217 (10)	−0.0006 (8)	−0.0001 (8)	−0.0014 (8)
C12	0.0148 (10)	0.0141 (10)	0.0199 (10)	0.0001 (8)	0.0033 (8)	−0.0030 (8)
C13	0.0203 (11)	0.0137 (10)	0.0138 (9)	−0.0050 (8)	0.0018 (8)	−0.0008 (8)
C14	0.0128 (10)	0.0147 (10)	0.0179 (10)	−0.0006 (8)	−0.0005 (8)	−0.0013 (8)
C15	0.0140 (10)	0.0143 (10)	0.0184 (10)	−0.0003 (8)	0.0023 (8)	−0.0027 (8)
C16	0.0228 (11)	0.0236 (12)	0.0172 (10)	−0.0034 (9)	−0.0027 (9)	−0.0022 (9)

### Geometric parameters (Å, °)

**Table:**

Cl1—C4	1.745 (2)	C8—H8A	0.9900
O1—C1	1.350 (2)	C8—H8B	0.9900
O1—H1	0.849 (10)	C9—C10	1.519 (3)
O2—C13	1.371 (2)	C9—H9A	0.9900
O2—C16	1.432 (3)	C9—H9B	0.9900
N1—C7	1.278 (2)	C10—C15	1.384 (3)
N1—C8	1.458 (2)	C10—C11	1.403 (3)
C1—C2	1.394 (3)	C11—C12	1.375 (3)
C1—C6	1.415 (3)	C11—H11	0.9500
C2—C3	1.382 (3)	C12—C13	1.395 (3)
C2—H2	0.9500	C12—H12	0.9500
C3—C4	1.390 (3)	C13—C14	1.388 (3)
C3—H3	0.9500	C14—C15	1.403 (3)
C4—C5	1.378 (3)	C14—H14	0.9500
C5—C6	1.395 (3)	C15—H15	0.9500
C5—H5	0.9500	C16—H16A	0.9800
C6—C7	1.463 (3)	C16—H16B	0.9800
C7—H7	0.9500	C16—H16C	0.9800
C8—C9	1.523 (3)		
			
C1—O1—H1	106.3 (19)	C10—C9—C8	115.68 (17)
C13—O2—C16	117.54 (16)	C10—C9—H9A	108.4
C7—N1—C8	120.28 (17)	C8—C9—H9A	108.4
O1—C1—C2	118.87 (17)	C10—C9—H9B	108.4
O1—C1—C6	121.37 (17)	C8—C9—H9B	108.4
C2—C1—C6	119.77 (17)	H9A—C9—H9B	107.4
C3—C2—C1	120.33 (18)	C15—C10—C11	117.62 (18)
C3—C2—H2	119.8	C15—C10—C9	124.08 (18)
C1—C2—H2	119.8	C11—C10—C9	118.28 (18)
C2—C3—C4	119.71 (18)	C12—C11—C10	121.49 (19)
C2—C3—H3	120.1	C12—C11—H11	119.3
C4—C3—H3	120.1	C10—C11—H11	119.3
C5—C4—C3	120.97 (18)	C11—C12—C13	120.15 (18)
C5—C4—Cl1	119.05 (15)	C11—C12—H12	119.9
C3—C4—Cl1	119.98 (15)	C13—C12—H12	119.9
C4—C5—C6	120.20 (18)	O2—C13—C14	125.16 (19)
C4—C5—H5	119.9	O2—C13—C12	115.10 (17)
C6—C5—H5	119.9	C14—C13—C12	119.73 (18)
C5—C6—C1	119.01 (17)	C13—C14—C15	119.15 (19)
C5—C6—C7	120.01 (17)	C13—C14—H14	120.4
C1—C6—C7	120.98 (17)	C15—C14—H14	120.4
N1—C7—C6	120.31 (17)	C10—C15—C14	121.83 (18)
N1—C7—H7	119.8	C10—C15—H15	119.1
C6—C7—H7	119.8	C14—C15—H15	119.1
N1—C8—C9	108.99 (16)	O2—C16—H16A	109.5
N1—C8—H8A	109.9	O2—C16—H16B	109.5
C9—C8—H8A	109.9	H16A—C16—H16B	109.5
N1—C8—H8B	109.9	O2—C16—H16C	109.5
C9—C8—H8B	109.9	H16A—C16—H16C	109.5
H8A—C8—H8B	108.3	H16B—C16—H16C	109.5
			
O1—C1—C2—C3	178.70 (19)	C7—N1—C8—C9	117.2 (2)
C6—C1—C2—C3	−1.0 (3)	N1—C8—C9—C10	159.86 (16)
C1—C2—C3—C4	0.7 (3)	C8—C9—C10—C15	13.5 (3)
C2—C3—C4—C5	0.2 (3)	C8—C9—C10—C11	−164.78 (17)
C2—C3—C4—Cl1	−179.87 (16)	C15—C10—C11—C12	−1.2 (3)
C3—C4—C5—C6	−0.9 (3)	C9—C10—C11—C12	177.17 (18)
Cl1—C4—C5—C6	179.22 (16)	C10—C11—C12—C13	−0.2 (3)
C4—C5—C6—C1	0.6 (3)	C16—O2—C13—C14	−2.5 (3)
C4—C5—C6—C7	−179.72 (19)	C16—O2—C13—C12	178.17 (17)
O1—C1—C6—C5	−179.34 (18)	C11—C12—C13—O2	−179.27 (18)
C2—C1—C6—C5	0.3 (3)	C11—C12—C13—C14	1.4 (3)
O1—C1—C6—C7	1.0 (3)	O2—C13—C14—C15	179.56 (18)
C2—C1—C6—C7	−179.37 (18)	C12—C13—C14—C15	−1.2 (3)
C8—N1—C7—C6	179.71 (17)	C11—C10—C15—C14	1.5 (3)
C5—C6—C7—N1	178.33 (19)	C9—C10—C15—C14	−176.86 (18)
C1—C6—C7—N1	−2.0 (3)	C13—C14—C15—C10	−0.3 (3)

### Hydrogen-bond geometry (Å, °)

**Table:**

*D*—H···*A*	*D*—H	H···*A*	*D*···*A*	*D*—H···*A*
O1—H1···N1	0.85 (1)	1.79 (2)	2.567 (2)	152 (3)
